# Adjustment for unmeasured confounding through informative priors for the confounder-outcome relation

**DOI:** 10.1186/s12874-018-0634-3

**Published:** 2018-12-22

**Authors:** Rolf H. H. Groenwold, Inbal Shofty, Milica Miočević, Maarten van Smeden, Irene Klugkist

**Affiliations:** 1 Department of Clinical Epidemiology, Leiden University Medical Center, Albinusdreef 2, 2333 ZA Leiden, The Netherlands; 2 Department of Biomedical Data Sciences, Leiden University Medical Center, Leiden, the Netherlands; 3 Julius Center for Health Sciences and Primary Care, University Medical Center Utrecht, Utrecht, The Netherlands; 4 Department of Methodology and Statistics, Faculty of Social and Behavioral Sciences, Utrecht University, Utrecht, The Netherlands; 5 Research Methodology, Measurement and Data Analysis of Behavioral, Management and Social Sciences, Twente University, Enschede, The Netherlands

**Keywords:** Bias, Confounding, Bayesian statistics, Sensitivity analysis

## Abstract

**Background:**

Observational studies of medical interventions or risk factors are potentially biased by unmeasured confounding. In this paper we propose a Bayesian approach by defining an informative prior for the confounder-outcome relation, to reduce bias due to unmeasured confounding. This approach was motivated by the phenomenon that the presence of unmeasured confounding may be reflected in observed confounder-outcome relations being unexpected in terms of direction or magnitude.

**Methods:**

The approach was tested using simulation studies and was illustrated in an empirical example of the relation between LDL cholesterol levels and systolic blood pressure. In simulated data, a comparison of the estimated exposure-outcome relation was made between two frequentist multivariable linear regression models and three Bayesian multivariable linear regression models, which varied in the precision of the prior distributions. Simulated data contained information on a continuous exposure, a continuous outcome, and two continuous confounders (one considered measured one unmeasured), under various scenarios.

**Results:**

In various scenarios the proposed Bayesian analysis with an correctly specified informative prior for the confounder-outcome relation substantially reduced bias due to unmeasured confounding and was less biased than the frequentist model with covariate adjustment for one of the two confounding variables. Also, in general the MSE was smaller for the Bayesian model with informative prior, compared to the other models.

**Conclusions:**

As incorporating (informative) prior information for the confounder-outcome relation may reduce the bias due to unmeasured confounding, we consider this approach one of many possible sensitivity analyses of unmeasured confounding.

**Electronic supplementary material:**

The online version of this article (10.1186/s12874-018-0634-3) contains supplementary material, which is available to authorized users.

## Background

Inferences from observational epidemiological studies are often hampered by confounding [1, 2]. To estimate the causal effect of exposure on the outcome, adjustment for a minimal set of confounding variables (or confounders) is required [3–6]. However, there may be unmeasured variables that result in unmeasured (or residual) confounding. Several design and analytical methods to account for unmeasured confounding have been proposed [7], including cross-over designs e.g., [8, 9], instrumental variable analysis e.g., [10, 11], the use of negative controls [12], and approaches to collect information on unmeasured confounding variables in a subsample e.g., [13, 14]. In addition, sensitivity analysis of unmeasured confounding is used to quantify the potential impact of unmeasured confounding [15–17].

Sensitivity analyses can be performed within a frequentist framework as well as within a Bayesian framework. The latter requires for example assumptions on prior distributions for the unknown parameters of the unmeasured confounder and its relations with exposure and outcome [18–21]. However, eliciting prior distributions for these unknown parameters can be very challenging as unmeasured confounders may actually be unknown. So far, Bayesian sensitivity analyses focused on allocating informative priors to the effect of the unmeasured confounders on the exposure or on the outcome [18, 19, 22]. Instead, it may be more straightforward to elicit prior distributions for the parameters of the effects of the observed confounders on the outcome.

Unmeasured confounding of the exposure-outcome relation may not only affect that relation, but may also bias the observed relations between confounders and outcome [23]. Constraining the estimation of the confounder-outcome relation, or incorporating (informative) prior information for the confounder-outcome relation, may (indirectly) reduce the bias due to unmeasured confounding of the exposure-outcome relation.

The aim of this research was to assess to what extent using prior information on parameters for an observed relation between a measured confounder and the outcome in a Bayesian analysis can reduce bias due to unmeasured confounding in an estimator of the exposure outcome relation. The remainder of this article is structured as follows. The bias due to omitting one or more confounders from a regression model is quantified in section 2. In section 3, the use of informative priors for the observed confounder-outcome relation was tested using simulation studies. Section 4 illustrates the approach using an empirical example of the relation between LDL cholesterol levels and systolic blood pressure. Section 5 provides a general discussion to the paper.

## Methods

### Notation

**Fig. 1:**
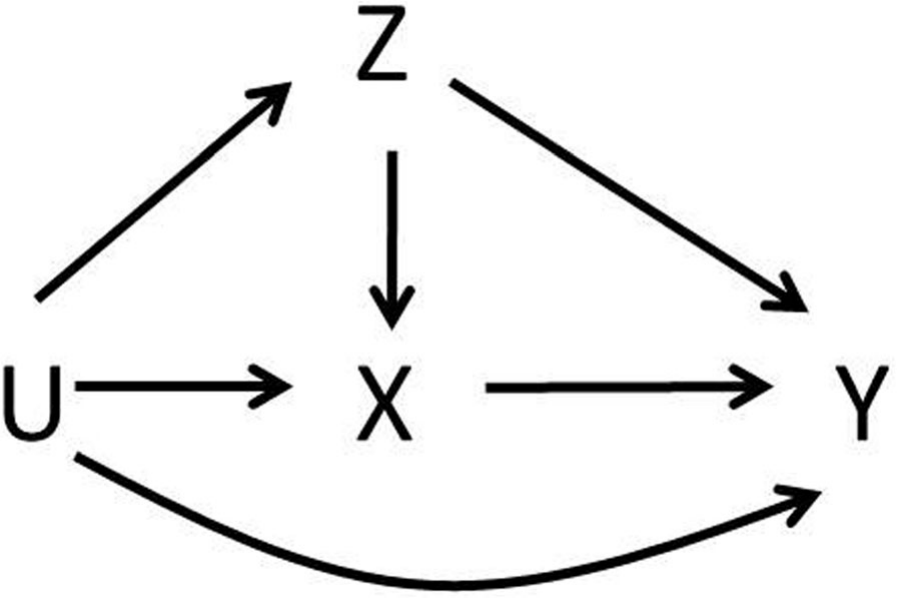
Structural relations between an exposure (X), an outcome (Y), and two confounders (Z and U) of the exposure-outcome relation. *See main text for details and explanation*

We consider studies of a continuous exposure (denoted by *X*), a continuous outcome (*Y*), and two continuous confounders (*Z* and *U*). All relations are assumed to be linear. All variables are considered related to the outcome, according to the model: *y*_*i*_ *= β*_*yx*_*x*_*i*_ *+ β*_*yz*_*z*_*i*_ *+ β*_*yu*_*u*_*i*_ *+ ε*_*i*_, where lower case letters represent the realisations of the random variables *Y*, *X*, *Z*, and *U*, *i* is a subject indicator (*i = 1, …, n*), and *ε ~ N(0,σ*^*2*^*)*. The confounders are considered related to the exposure: *x*_*i*_ *= β*_*xz*_*z*_*i*_ *+ β*_*xu*_*u*_*i*_ *+ ζ*_*i*_, and the confounders are also related to each other: *z*_*i*_ *= β*_*zu*_*u*_*i*_ *+ ξ*_*i*_, with *ζ ~ N(0,σ*_*x*_^*2*^*)* and *ξ ~ N(0,σ*_*z*_^*2*^*)*. For all models, the intercepts are assumed independent of all other terms in the models and are omitted here and in the following equations. The coefficients of these models represent an increase in the dependent variable by *β*_*..*_ for each unit increase in the independent variable. The structural relations between the variables are presented in Fig. 1.

### Bias due to unmeasured confounding

For the fairly simple model outlined in Fig. 1, there are three possible scenarios of confounding adjustment: scenario 1.) both confounders *Z* and *U* are measured and adjusted for (e.g., by a multivariable regression analysis of *Y* on *X*, including *Z* and *U* as covariates); scenario 2.) none of the confounders are measured and hence none is adjusted for; and scenario 3.) one confounder (*Z*) is measured and adjusted for, while the other (*U*) is not. Because our interest is in situations in which unmeasured confounding is present, we only consider scenarios 2 and 3.

In both scenarios, the effect of *X* on *Y* can be estimated by means of a linear regression model. In the following, we assume all assumptions of the linear regression model are met, except that unmeasured confounding may be present. As a result, the estimator for the effect of *X* on *Y* is expected to be biased due to unmeasured confounding. Details about the bias due to unmeasured confounding are provided in Additional file 1: Appendix 1.

In scenario 2, the bias due to omitting *Z* and *U* from the data analytical model can be expressed as:

1 $$ bias\left({\beta}_{yx}\right)={\beta}_{yz}\left({\beta}_{xz}\frac{Var(Z)}{Var(X)}+{\beta}_{zu}{\beta}_{xu}\frac{Var(U)}{Var(X)}\right)+{\beta}_{yu}\frac{Var(U)}{Var(X)}\left({\beta}_{xu}+{\beta}_{zu}{\beta}_{xz}\right), $$

where *Var(Z)*, *Var(X),* and *Var(U)* denote the marginal variances of *Z*, *X*, and *U*, respectively. Equation (1) indicates that the bias resulting from omitting two confounders is independent of the true exposure-outcome relation *β*_*yx*_. Furthermore, the bias increases with increasing strength of the relation between each of the confounders and the outcome or the exposure (*β*_*yz*_*, β*_*yu*_*, β*_*xz*_*,* and *β*_*xu*_). The bias is the result of different backdoor paths [24] from *X* to *Y*: X ← Z → Y, X ← U → Y, X ← Z ← U → Y, and X ← U → Z → Y, which can be identified in the equation.

In scenario 3 the bias due to omitting *U* from the data analytical model, while adjusting for *Z*, can be expressed as:

2 $$ bias\kern0.5em \left({\beta}_{yx\mid z}\right)\kern0.5em =\kern0.5em {\beta}_{xu}{\beta}_{yu}\frac{Var(U)\left(1-{\rho}_{uz}^2\right)}{Var(X)\left(1-{\rho}_{xz}^2\right)}, $$

where ${\rho}_{uz}^2$ is the squared (Pearson’s) correlation between *U* and *Z*, ${\rho}_{xz}^2$ is the squared correlation between *X* and *Z*, and $Var(U)\left(1-{\rho}_{uz}^2\right)$ and $Var(X)\left(1-{\rho}_{xz}^2\right)$, represent the conditional variances of *U* given *Z* and of *X* given *Z*, respectively. Equation (2) shows that the bias resulting from omitting one confounder from the adjustment model is independent of the true exposure-outcome relation *β*_*yx*_. Furthermore, the bias increases as the relation between the unmeasured confounder and the outcome (*β*_*yu*_) or the exposure (*β*_*xu*_) increases.

As the correlation between the confounders (*ρ*_*uz*_) increases, the bias of the estimator of the exposure-outcome relation decreases. Intuitively, when two confounders are correlated, adjusting for one accounts for some of the variability (and thus confounding effect) in the other. Therefore, adjustment for one confounder may reduce the bias that is caused by the other [25, 26]. In addition, in a linear model, *Var(X|Z) ≤ Var(X)* and the larger the absolute value of *ρ*_*xz*_ the smaller *Var(X|Z)*. Because of this decreased *Var(X|Z)*, the residual bias carried by *U*, i.e.${\beta}_{xu}{\beta}_{yu} Var(U)\left(1-{\rho}_{uz}^2\right)$, is amplified. This bias amplification particularly happens when the confounder (*Z*) that is adjusted for acts like an instrumental variable (IV) or near-IV, meaning that it has a stronger relation with the exposure (*X*) than with the outcome (*Y*) [27, 28].

In scenario 3, the linear regression analysis of *Y* on *X* and *Z*, yielding an estimate of *β*_*yx* ∣ *z*_, is a biased estimator of the relation between *X* and *Y*. However, this linear regression analysis is also a biased estimator of the relation between *Z* and *Y* (*β*_*yz* ∣ *x*_). When we assume all variables follow a multivariate standard normal distribution, the bias in the *β*_*yz* ∣ *x*_ relation can be expressed as:

3 $$ bias\left({\beta}_{yz\mid x}\right)\kern0.5em =\kern0.5em {\beta}_{yu}^{\hbox{'}}\left(\frac{\rho_{zu}-{\rho}_{xz}{\rho}_{xu}}{1-{\rho}_{xz}^2}\right), $$

where ${\beta}_{yu}^{\prime }$ represents the conditional (or direct) effect of *U* on *Y* if both are standardized. Equation (3) shows that the unmeasured confounder (*U*) of the exposure-outcome relation may also confound the observed relation between the measured confounder (*Z*) and the outcome. If *Z* and *X* are independent (i.e., *ρ*_*xz*_ *= 0*), the bias is simply the result of the backdoor path from *Z* to *Y* via *U* (i.e., ${\beta}_{yu}^{\prime }{\rho}_{zu}$). Note that even if *Z* and *U* are independent, the observed relation between *Z* and *Y* is biased, due to conditioning on *X*, which is a collider of *Z* and *U* and hence conditioning on *X* opens a path from *Z* to *Y* via *U* [24].

### Reducing unmeasured confounding using a Bayesian model

As indicated above, unmeasured confounding of the exposure-outcome relation can also bias the relation between an observed confounder and the outcome. Hence, an unexpected relation between a confounder and the outcome may suggest the presence of unmeasured confounding. Allocating informative priors to the observed confounder-outcome relation may not only reduce the bias in that parameter, but also may reduce the bias due to unmeasured confounding of the exposure-outcome relation.

In the absence of information about the confounder *U*, the relation between *X* and *Y* only can be controlled for confounding by *Z*. In a Bayesian framework, we can specify a linear model of *Y* as a function of *X* and *Z*. The parameters of interest, *β*_*yx*_, *β*_*yz*_ and *σ*^*2*^, can then be estimated using their joint posterior distribution given the data for *Y*, *X*, and *Z*. The joint posterior distribution is proportional to the product of the density of the data times the joint prior distribution of the parameters:

4 $$ P\left({\beta}_{yx},{\beta}_{yz},{\sigma}^2|Y,X,Z\right)\alpha f\left(Y|X,Z,{\beta}_{yx},{\beta}_{yx},{\sigma}^2\right)g\left({\beta}_{xy},{\beta}_{yz},{\sigma}^2\right), $$

where *g*(*β*_*xy*_, *β*_*yz*_, *σ*^2^) is the joint prior distribution and *f*(*Y*| *X*, *Z*, *β*_*yx*_, *β*_*yz*_, *σ*^2^) is the probability density of *Y* conditional on the parameters:

5 $$ f\left(Y|X,Z,{\beta}_{yx},{\beta}_{yz},{\sigma}^2\right)={\prod}_i\frac{1}{\sqrt{2{\pi \sigma}^2}}\exp \left(\frac{-{\left({y}_i-{\beta}_{yx}{x}_i-{\beta}_{yz}{z}_i\right)}^2}{2{\sigma}^2}\right). $$

Assuming independent priors for the different parameters, the joint prior is simply a product of all marginal priors.

Incorporating (informative) prior information for the confounder-outcome relation, may (indirectly) reduce the bias due to unmeasured confounding (by the unmeasured variable *U*) of the exposure-outcome relation. This was tested through simulation studies, which are described in the next section.

### Simulation study of Bayesian analysis to control for unmeasured confounding

#### Objective

A simulation study was performed to test the possible decrease in bias in the estimator of the exposure-outcome relation by using informative priors for the confounder-outcome relation. In simulated data, a comparison of the estimated relation between the exposure (*X*) and the outcome (*Y*) was made between two frequentist (OLS) multivariable linear regression models and three Bayesian multivariable linear regression models.

#### Data analysis

Every simulated data set was analysed in five different ways: two frequentist analyses and three Bayesian analyses. The two frequentist regression models included none or one of the two confounding variables: linear regression analysis without and with adjustment for the measured confounder Z. The three Bayesian regression analyses all incorporated the information about one confounder, but used different informative priors for the confounder-outcome relation. The performance of these methods was compared in terms of bias and precision of the estimator of the exposure-outcome relation. The simulation study was performed in R, version 3.1.1 [29].

The Bayesian model described in section 2.3 was used. All Bayesian regression analyses were adjusted for *Z*, but not for *U*. We used uninformative priors for *σ*^*2*^ and *β*_*yx*_: *σ ∼ U(0,100)* and *β*_*yx*_ *∼ N(μ = 0, τ = 0.001)*, where τ indicates the precision of the distribution. We used informative priors for the parameter *β*_*yz*_, but with different levels of precision. A normal informative prior was assumed for *β*_*yz*_, with the true value for *β*_*yz*_ as the mean and different values for the precision, which were proportionate to the sample size *n* of the simulated data sets: *β*_*yz*_ *∼ N(μ = β*_*yz*_*, τ = n, n/10, n/100)*. The precision could take three different values representing different degrees of certainty in the prior information. The Bayesian models were specified using the rjags package in R [30], which provides an interface from R to JAGS (http://mcmc-jags.sourceforge.net).

Since the priors for *σ*_*y*_ and *β*_*yx*_ were non-informative, the posterior distributions could be approximated by the product of the density of the data and the prior of *β*_*yz*_. The Gibbs sampler was used with four parallel chains for 2000 iterations. The first 1000 iterations were discarded as burn-in runs. Since the marginal posterior was normal, we chose to present the mean of the posterior distribution as an estimate of *β*_*yx|z*_.

#### Data generation

Data were generated according to the structure depicted in Fig. 1 and consisted of a continuous exposure (*X*), a continuous outcome (*Y*), and two continuous confounders (*Z* and U). First, U was sampled from a normal distribution: *U ~ N(0, σ*_*u*_^*2*^*)*. Second, *Z* was generated based on *U*: *z*_*i*_ *= β*_*zu*_*u*_*i*_ *+ ξ*_*i*_, with *ξ ~ N(0, σ*_*z*_^*2*^*)*. Then, *X* was generated based on *U* and *Z*: *x*_*i*_ *= β*_*xz*_*z*_*i*_ *+ β*_*xu*_*u*_*i*_ *+ ζ*_*i*_, with *ζ ~ N(0, σ*_*x*_^*2*^*)*. Finally, *Y* was generated based on *U*, *Z*, and *X*: *y*_*i*_ *= β*_*yx*_*x*_*i*_ *+ β*_*yz*_*z*_*i*_ *+ β*_*yu*_*u*_*i*_ *+ ε*_*i*_, with *ε ~ N(0, σ*^*2*^*)*.

In all simulations, the variances *σ*_*u*_^*2*^, *σ*_*z*_^*2*^, *σ*_*x*_^*2*^, and *σ*^*2*^ were set to 1. Furthermore, the exposure-outcome relation was fixed at *β*_*yx*_ *= 0* (i.e. zero relation). The parameter *β*_*zu*_ was set at 0, or 1. The parameters *β*_*yz*_, and *β*_*xz*_ were set at 1 or 2, indicating that the observed confounder *Z* was related to *X* and to *Y* in all scenarios. The parameters *β*_*yu*_ and *β*_*xu*_ were set at 0, 1, or 2. All combinations of the parameters settings were evaluated through simulations, leading to 72 different scenarios.

#### Comparison of methods

For each scenario 100 datasets of 1000 subjects each were generated. In each dataset the methods described above were applied. For each scenario separately, the performance of these methods was compared in terms of bias of the estimator of the relation between *X* and *Y*, the empirical standard deviation (SD) of the estimated relations between *X* and *Y*, and the mean squared error (MSE). For the frequentist models, we computed the average of the estimated regression coefficients (bias), their standard deviation (SD), and the mean of the squared difference between the estimated regression coefficient and the true exposure-outcome relation (MSE). For the Bayesian models, we computed the average of the posterior means (bias), their standard deviation (SD), and the mean of the squared difference between the posterior mean and the true exposure-outcome relation (MSE).

### Example study of the relation between cholesterol levels and blood pressure

To illustrate the application of the use of informative priors for the observed confounder-outcome relation we used data on the relation between low-density lipoprotein (LDL cholesterol) levels and systolic blood pressure (SBP). This example was based on the Second Manifestations of Arterial disease (SMART) study, which is an ongoing prospective cohort study of patients with manifest vascular disease of vascular risk factors [31]. For this example, we assumed that there are two possible confounders of the LDL-SBP relation, namely body mass index (BMI) and blood glucose levels (BGL). A data set of 1000 observations was simulated based on the variance-covariance matrix and the vector of means of these four variables in the cohort study. In all analyses, BMI was considered to be a measured confounder, while BGL was considered to be unmeasured.

#### Comparison of methods

The different methods described in section 3.2.1 were applied to the example data. As a reference, we fitted a linear regression model of SBP on LDL, including BMI and BGL as covariates (referred to as the ‘full model’). BMI was considered to be a measured confounder, while BGL was considered to be unmeasured. The performance of the different methods was assessed by the difference between the estimated LDL-SBP relations from the different models and the LDL-SBP relation obtained from the full model.

The Bayesian approach was implemented in two ways. We first used the estimated regression coefficient of the effect of BMI on systolic blood pressure from the full model (i.e., 0.32), as the mean for the prior distribution of the measured confounder on the outcome, and precision equal to the sample size (i.e., τ = 1000). We then used an relation from the literature as the prior mean. A previous study on the relation between BMI and SBP in adults found a linear regression coefficient of 0.77 [32]. This relation was used as the mean of the prior distribution of the measured confounder and outcome. Since we were less certain about this prior information, we used a smaller precision (τ = 100). For all the other relations we used uninformative priors as described in Section 3.2.1.

## Results

### Simulation study

Table 1 shows the results of the simulation study for the scenarios where *β*_*xz*_ *= β*_*yz*_ *= 2*. Similar patterns were observed for other values of *β*_*xz*_ and *β*_*yz*_; these are omitted from the Table for brevity. Results for all simulated scenarios can be found in Additional file 2: Appendix 2. The Bayesian model with precision 100 (i.e., n/10) showed results that were in between those of the Bayesian models with precision 1000 (i.e., n) and precision 10 (i.e., n/100). Results for the Bayesian model with precision 100 are omitted for clarity (see Additional file 2: Appendix 2).

**Table 1:** Results of the simulation study of different methods to control for confounding

Scenario	Parameter settings				Frequentist model						Bayesian model					
					Unadjusted			Adjusted for Z			Adjusted for Z, τ = 1000			Adjusted for Z, τ = 10		
	*β*_*zu*_	*β*_*xz*_	*β*_*xu*_	*β*_*yu*_	Bias	SD	MSE	Bias	SD	MSE	Bias	SD	MSE	Bias	SD	MSE
1	0	1	0	0	0.50	0.027	0.25	0.00	0.034	0.0011	0.00	0.025	0.0006	0.00	0.033	0.0011
2	1	1	0	0	0.67	0.027	0.45	0.00	0.035	0.0012	0.00	0.022	0.0005	0.00	0.034	0.0012
3	0	1	1	0	0.33	0.024	0.11	0.00	0.024	0.0006	0.00	0.021	0.0004	0.00	0.024	0.0006
4	1	1	1	0	0.50	0.016	0.25	0.00	0.031	0.0009	0.00	0.017	0.0003	0.00	0.030	0.0009
5	0	1	2	0	0.17	0.017	0.029	0.00	0.014	0.0002	0.00	0.013	0.0002	0.00	0.014	0.0002
6	1	1	2	0	0.36	0.012	0.13	0.00	0.020	0.0004	0.00	0.011	0.0001	0.00	0.020	0.0004
7	0	1	0	1	0.50	0.034	0.25	0.00	0.043	0.0019	0.00	0.032	0.001	0.00	0.043	0.0018
8	1	1	0	1	1.00	0.036	1.00	0.00	0.034	0.0012	0.23	0.026	0.055	0.00	0.034	0.0012
9	0	1	1	1	0.67	0.023	0.45	0.50	0.029	0.25	0.38	0.024	0.15	0.50	0.029	0.25
10	1	1	1	1	0.83	0.018	0.69	0.33	0.028	0.11	0.33	0.017	0.11	0.33	0.028	0.11
11	0	1	2	1	0.50	0.016	0.25	0.40	0.016	0.16	0.36	0.015	0.13	0.40	0.015	0.16
12	1	1	2	1	0.64	0.011	0.41	0.33	0.018	0.11	0.29	0.010	0.085	0.33	0.017	0.11
13	0	1	0	2	0.50	0.049	0.25	−0.01	0.066	0.0044	0.00	0.047	0.0022	−0.01	0.063	0.004
14	1	1	0	2	1.34	0.045	1.79	0.01	0.057	0.0033	0.57	0.036	0.32	0.034	0.055	0.0042
15	0	1	1	2	1. 01	0.032	1.00	1.00	0.037	1.00	0.72	0.035	0.52	0.99	0.037	0.97
16	1	1	1	2	1.17	0.024	1.36	0.67	0.038	0.45	0.67	0.021	0.45	0.67	0.037	0.45
17	0	1	2	2	0.83	0.019	0.70	0.80	0.020	0.64	0.71	0.019	0.50	0.80	0.020	0.64
18	1	1	2	2	0.91	0.013	0.83	0.67	0.023	0.45	0.58	0.012	0.33	0.66	0.022	0.44

Bias refers to the bias in the estimator of the relation between X and Y, compared to the true X-Y relation (*βyx = 0*). τ indicates the precision of the prior distribution of the Z-Y relation in the Bayesian model and is proportional to the sample size of each generated data set (*n* = 1000). Abbreviations: *SD* – standard deviation of the empirical distributions of the parameter estimates; *MSE* – mean squared error of the parameter estimates. See text for details on simulation study

In most scenarios, the Bayesian model with precision 1000 showed less bias than the frequentist model with covariate adjustment. Noticeable exceptions in Table 1 are scenarios 8 and 14, in which the Bayesian model with precision 1000 was more biased than the frequentist model with covariate adjustment (which was actually unbiased). The reason for this is that in these scenarios *U* is not a confounder of the *X-Y* relation (because β_xu_ = 0), yet it is a confounder of the *Z-Y* relation (e.g., in scenario 8 $\widehat{\beta_{yz\mid x}}$= 1.50, while β_yz_ = 1). As the Bayesian model corrects the bias in the *Z-Y* relation, it induces a bias in the *X-Y* relation. In scenarios 10 and 16 in Table 1, the Bayesian models and the frequentist model with covariate adjustment yielded similar, yet biased, results. In these scenarios, the estimated relation between *Z* and *Y* from the frequentist model with covariate adjustment corresponded with the mean of the prior distribution of this relation (i.e., $\widehat{\beta_{yz\mid x}}$= 1.00 and β_yz_ = 1). Hence, the Bayesian model did not reduce bias, compared to the frequentist model. In scenarios 1–7, all methods that adjusted for the measured confounder *Z* yielded unbiased results, because the variable *U* was not a confounder in these scenarios (β_yu_ = 0). The extent to which the Bayesian model reduced bias was substantially smaller when the precision was 10 instead of 1000.

The standard deviation (SD) of the empirical distribution of the parameter estimates was smaller for the Bayesian model with precision 1000, compared to the frequentist model with covariate adjustment and the Bayesian model with precision 10 (the latter two showing approximately the same SD). Also, in general MSE was smaller for the Bayesian model with precision 1000, compared to the other models.

### Empirical example

In the empirical example of the relation between low-density lipoprotein (LDL cholesterol) levels and systolic blood pressure (SBP)., LDL increased BP, after adjustment for BMI and BGL, but omitting BGL from the data analytical model reduced the estimated effect substantially from 1.24 to 1.03 (Table 2). The amount of bias of the LDL-SBP relation slightly decreased when using an informative prior for the confounder outcome relation (i.e., for the BMI-SBP relation). However, even when the ‘correct’ prior, based on the full model, was used, the estimated effect of LDL on SBP remained substantially different from the reference value.

**Table 2:** Estimated effect of LDL cholesterol levels on systolic blood pressure, using different methods to deal with unmeasured confounding

	Reference^a^	Frequentist analysis	Bayesian analysis - 1	Bayesian analysis - 2
Prior for relation BMI-SBP^b^	–	–	N(μ = 0.32, τ = 1000)	N(μ =0.77, τ = 100)
Estimated effect of LDL on SBP^b^	1.24 (0.53)	1.03 (0.53)	1.06 (0.53)	1.05 (0.54)
Estimated effect of BMI on SBP^b^	0.32 (0.15)	0.44 (0.14)	0.33 (0.03)	0.66 (0.08)

Figures represent estimates (SE) of the estimated relations, or the mean (standard deviation) of the posterior distributions. In all analyses (except for the reference), BMI was considered a measured confounder of the LDL-SBP relation, while blood glucose level was considered unmeasured confounder. Bayesian analysis 1 and Bayesian analysis 2 differ in the mean and precision of the prior distribution of the relation between BMI and SBP

^a^The reference is based on the full model, i.e., is adjusted for BMI and blood glucose levels

^b^SBP was measured in mmHg, LDL in mmol/l, and BMI in kg/m^2^

## Discussion

This simulation study on the value of Bayesian analysis with informative priors for the relation between the measured confounder and the outcome in the presence of unmeasured confounding shows that such an analysis can reduce the bias due to unmeasured confounding substantially. The magnitude of the remaining bias decreases as the precision of the (correct) informative prior increases.

An obvious prerequisite when using the proposed Bayesian approach to correct for unmeasured confounding is prior knowledge about the relation between the measured confounder and the outcome. We argue that in many clinical research situations, such prior knowledge exists for many observed confounders, at least in terms of direction and order of magnitude of the relation. That information may be obtained from rigorously designed and conducted large epidemiological studies or from meta-analysis of individual patient data of randomised trials. Obviously, the impact of the Bayesian approach depends on the precision of the prior distribution. Informative priors with relatively small precision have little impact in term of confounding correction, yet allow Bayesian algorithms to be used. In practice it might be difficult – or researchers may be reluctant – to specify relatively highly informative priors.

If only the direction (but not the magnitude) of the confounder-outcome relation is included in the prior, the precision of the prior will be relatively small and the impact of the Bayesian analysis may be relatively small too. We did not include this particular form of prior distribution in our simulation study, but instead focused on distributions with the same mean, yet different precision.

As with any simulation study, an obvious limitation to our work is the finite number of simulated scenarios that we evaluated. For example, we only considered situations with two confounders, one being measured, one unmeasured. Although the two confounders Z and U could be considered as representing two sets of measured and unmeasured confounders, respectively, future research could address scenarios of multiple confounders with, e.g., different distributions of the confounders. Another scenario that we did not evaluate and could be the topic of future research is specification of the priors, such that these do not correspond to the ‘true’ confounder-outcome relation. The robustness to various levels of misspecifications of the prior distribution still needs to be studied.

Where to position this Bayesian approach in the toolbox of the researcher doing observational epidemiologic research? Given that many observational studies potentially suffer from unmeasured confounding, sensitivity analysis of unmeasured confounding is often important. Eliciting priors for unobserved (and possibly unknown) confounding variables is likely to be difficult. On the other hand, focusing on the approximate size of the relations between *measured* confounders and the outcome provides the opportunity to perform a Bayesian sensitivity analysis as outlined in this paper.

Informative priors for the measured confounder-outcome relations can reduce unmeasured confounding bias of the exposure-outcome relation. In case of observing unexpected confounder-outcome relations a sensitivity analysis of unmeasured confounding could be considered, in which prior information about the observed confounder-outcome relations is incorporated through Bayesian analysis.

## Conclusions

In this paper we proposed a Bayesian approach to reduce bias due to unmeasured confounding by expressing an informative prior for a measured confounder-outcome relation. A simulation study on the value of this Bayesian analysis with informative priors for the relation between the measured confounder and the outcome in the presence of unmeasured confounding shows that such an analysis can indeed reduce the bias due to unmeasured confounding substantially. The magnitude of the remaining bias decreases as the precision of the (correct) informative prior increases. We consider this approach one of many possible sensitivity analyses of unmeasured confounding.

## Additional files

Additional file 1: Appendix 1. Expressions of bias. (PDF 105 kb)

Additional file 2: Appendix 2 Table A1. Results of the simulation study of different methods to control for confounding (PDF 395 kb)

## Abbreviations

BGL: Blood glucose levels
BMI: Body mass index
LDL: Low-density lipoprotein
MSE: Mean squared error
SBP: Systolic blood pressure
SD: Standard deviation
