# Supplementary material for: Adjustment for unmeasured confounding through informative priors for the confounder-outcome relation
Source: BMC Med Res Methodol. 2018 Dec 22;18:174. doi: 10.1186/s12874-018-0634-3 (PMC6303957; doi:10.1186/s12874-018-0634-3)
Supplement: Supplementary file 1 — Appendix 1. Expressions of bias. (PDF 105 kb) [file 12874_2018_634_MOESM1_ESM.pdf]

Appendix 1

In the following, we consider the model presented in Figure A1 as the data generating mechanism. See main text for details on the model and notation.

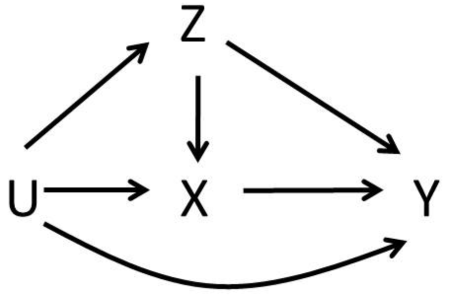

Figure A1: Structural relations between an exposure (X), an outcome (Y), and two confounders (Z and U) of the exposure-outcome relation.

Bias due to two unmeasured confounders

Let D be the design matrix, which in case of two unmeasured confounders is simply the vector matrix X. The estimator of the relation between X and Y can then be expressed as:

$$\widehat{\beta_{yx}} = (D^{'}D)^{-1}D^{'}Y.$$

Since $E[Y] = X\beta_{yx} + Z\beta_{yz} + U\beta_{yu}$, $E[Z] = X\beta_{xz}\frac{Var(Z)}{Var(X)} + \beta_{zu}\beta_{xu}\frac{Var(U)}{Var(X)}$, and $E[U] = X\beta_{yu}\frac{Var(U)}{Var(X)}(\beta_{xu} + \beta_{zu}\beta_{xz}$, it follows that the expected value of the total effect of X on Y based on a model without U and Z is given by:

$$E\left[\widehat{\beta_{yx}}\right] = \beta_{yx} + \beta_{yz}(\beta_{xz}\frac{Var(Z)}{Var(X)} + \beta_{zu}\beta_{xu}\frac{Var(U)}{Var(X)}) + \beta_{yu}\frac{Var(U)}{Var(X)}(\beta_{xu} + \beta_{zu}\beta_{xz})$$

Hence, the bias in the OLS estimator of the relation between X and Y, when omitting U and Z is given by:

$$bias(\beta_{yx}) = \beta_{yz}(\beta_{xz}\frac{Var(Z)}{Var(X)} + \beta_{zu}\beta_{xu}\frac{Var(U)}{Var(X)}) + \beta_{yu}\frac{Var(U)}{Var(X)}(\beta_{xu} + \beta_{zu}\beta_{xz}) \quad (1)$$

Bias due to one unmeasured confounder

When Z is a measured confounder, while U is unmeasured, the design matrix D is the two column matrix of X and Z.

The expected value of the estimated total effect of X on Y, when adjusting for Z (but without adjustment for U) is then given by the first element of $(\boldsymbol{D}'\boldsymbol{D})^{-1}\boldsymbol{D}'\boldsymbol{Y}$:

$$\begin{aligned}\hat{\boldsymbol{\beta}} &= (\boldsymbol{D}'\boldsymbol{D})^{-1}\boldsymbol{D}'\boldsymbol{Y} \\ &= \begin{pmatrix} \boldsymbol{Z'ZX'Y - X'ZZ'Y} \\ \boldsymbol{-Z'XX'Y + X'XZ'Y} \end{pmatrix} \frac{\boldsymbol{1}}{\boldsymbol{X'XZ'Z - X'ZZ'X}}.\end{aligned}$$

The first row of $\hat{\boldsymbol{\beta}}$ corresponds to the estimated effect of X on Y and the second row to the effect of Z on Y. Using the same arguments as before, we can derive an expression for the expected value of the total effect of X on Y, conditional on Z ($\boldsymbol{\beta_{yx|z}}$), but ignoring U:

$$\boldsymbol{E}[\widehat{\boldsymbol{\beta_{yx|z}}}] = \boldsymbol{\beta_{yx}} + \boldsymbol{\beta_{xu}}(\boldsymbol{\beta_{yu}}\frac{\boldsymbol{Var(U)(1-\rho^2_{zu})}}{\boldsymbol{Var(X)(1-\rho^2_{xz})}}, \tag{2}$$

where $\boldsymbol{\rho_{zu}}$ and $\boldsymbol{\rho_{xz}}$ are the (marginal) correlations between Z and U and between X and Z, respectively. The term $\boldsymbol{Var(U)(1-\rho zu^2)}$ represents the conditional variance of U, given Z. The term $\boldsymbol{Var(X)(1-\rho xz^2)}$ represents the conditional variance of X, given Z.

The expected value of the estimated effect of Z on Y, conditional on X, is given by the second row of $\hat{\boldsymbol{\beta}}$. The expression for the expected value of the effect of Z on Y, conditional on X ($\boldsymbol{\beta_{yz|x}}$), but ignoring U is given by:

$$\boldsymbol{E}[\widehat{\boldsymbol{\beta_{yz|x}}}] = \boldsymbol{\beta'_{yu}}(\frac{\boldsymbol{\rho_{zu} - \rho_{xz}\rho_{xu}}}{\boldsymbol{1-\rho^2_{xz}}}), \tag{3}$$

where $\boldsymbol{\beta'_{yu}}$ represents the conditional (or direct) effect of U on Y, if both are standardized. $\boldsymbol{\rho}$ again represents (marginal) correlations.
