# Supplementary material for: Adjustment for unmeasured confounding through informative priors for the confounder-outcome relation
Source: BMC Med Res Methodol. 2018 Dec 22;18:174. doi: 10.1186/s12874-018-0634-3 (PMC6303957; doi:10.1186/s12874-018-0634-3)
Supplement: Supplementary file 2 — Appendix 2 Table A1. Results of the simulation study of different methods to control for confounding (PDF 395 kb) [file 12874_2018_634_MOESM2_ESM.pdf]

APPENDIX 2

Table A1. Results of the simulation study of different methods to control for confounding

Parameter settings					Frequentist model						Bayesian model								
					Unadjusted			*Adjusted for Z*			*Adjusted for Z, τ = 1000*			*Adjusted for Z, τ = 100*			*Adjusted for Z, τ = 10*		
β_{zu}	β_{xz}	β_{xu}	β_{yz}	β_{yu}	***Bias***	***SD***	***MSE***	***Bias***	***SD***	***MSE***	***Bias***	***SD***	***MSE***	***Bias***	***SD***	***MSE***	***Bias***	***SD***	***MSE***
0	1	0	1	0	0.5	0.027	0.2510	0.00	0.034	0.0011	0.00	0.025	0.0006	0.00	0.031	0.0009	0.00	0.033	0.0011
1	1	0	1	0	0.67	0.027	0.4533	0.00	0.035	0.0012	0.00	0.022	0.0005	0.00	0.031	0.001	0.00	0.034	0.0012
0	2	0	1	0	0.4	0.017	0.1607	0.00	0.028	0.0008	0.00	0.016	0.0003	0.00	0.022	0.0005	0.00	0.027	0.0007
1	2	0	1	0	0.45	0.011	0.1991	0.00	0.032	0.0010	0.00	0.013	0.0002	0.00	0.024	0.0006	0.00	0.031	0.0010
0	1	1	1	0	0.33	0.024	0.1118	0.00	0.024	0.0006	0.00	0.021	0.0004	0.00	0.023	0.0005	0.00	0.024	0.0006
1	1	1	1	0	0.5	0.016	0.2484	0.00	0.031	0.0009	0.00	0.017	0.0003	0.00	0.027	0.0007	0.00	0.030	0.0009
0	2	1	1	0	0.33	0.015	0.1120	0.00	0.022	0.0005	0.00	0.013	0.0002	0.00	0.019	0.0003	0.00	0.022	0.0005
1	2	1	1	0	0.36	0.009	0.1291	0.00	0.028	0.0008	0.00	0.010	0.0001	0.00	0.020	0.0004	0.00	0.027	0.0007
0	1	2	1	0	0.17	0.017	0.0286	0.00	0.014	0.0002	0.00	0.013	0.0002	0.00	0.013	0.0002	0.00	0.014	0.0002
1	1	2	1	0	0.36	0.012	0.1318	0.00	0.020	0.0004	0.00	0.011	0.0001	0.00	0.018	0.0003	0.00	0.020	0.0004
0	2	2	1	0	0.22	0.013	0.0499	0.00	0.015	0.0002	0.00	0.011	0.0001	0.00	0.014	0.0002	0.00	0.015	0.0002
1	2	2	1	0	0.29	0.008	0.0817	0.00	0.020	0.0004	0.00	0.009	0.0001	0.00	0.016	0.0003	0.00	0.019	0.0004
0	1	0	2	0	1.00	0.041	1.0009	0.00	0.034	0.0012	0.00	0.027	0.0007	0.00	0.032	0.0010	0.00	0.034	0.0012
1	1	0	2	0	1.34	0.034	1.7879	0.00	0.031	0.0010	0.00	0.020	0.0004	0.00	0.028	0.0008	0.00	0.031	0.0009
0	2	0	2	0	0.8	0.021	0.6427	0.00	0.036	0.0013	0.00	0.017	0.0003	0.00	0.027	0.0007	0.00	0.035	0.0012
1	2	0	2	0	0.89	0.014	0.7924	0.00	0.028	0.0008	0.00	0.011	0.0001	0.00	0.021	0.0004	0.00	0.027	0.0007
0	1	1	2	0	0.67	0.033	0.4440	0.00	0.022	0.0005	0.00	0.019	0.0004	0.00	0.021	0.0005	0.00	0.022	0.0005
1	1	1	2	0	1.00	0.024	1.0036	0.00	0.028	0.0008	0.00	0.017	0.0003	0.00	0.025	0.0006	0.00	0.028	0.0008
0	2	1	2	0	0.67	0.021	0.4433	0.00	0.024	0.0006	0.00	0.015	0.0002	0.00	0.021	0.0004	0.00	0.024	0.0006
1	2	1	2	0	0.71	0.012	0.5102	0.00	0.027	0.0007	0.00	0.010	0.0001	0.00	0.020	0.0004	0.00	0.026	0.0007
0	1	2	2	0	0.34	0.029	0.1163	0.00	0.014	0.0002	0.00	0.013	0.0002	0.00	0.013	0.0002	0.00	0.013	0.0002
1	1	2	2	0	0.73	0.018	0.5264	0.00	0.019	0.0004	0.00	0.012	0.0001	0.00	0.017	0.0003	0.00	0.019	0.0004
0	2	2	2	0	0.45	0.020	0.1991	0.00	0.013	0.0002	0.00	0.011	0.0001	0.00	0.012	0.0002	0.00	0.013	0.0002
1	2	2	2	0	0.57	0.010	0.3257	0.00	0.018	0.0003	0.00	0.007	0.0001	0.00	0.014	0.0002	0.00	0.017	0.0003
0	1	0	1	1	0.50	0.034	0.2509	0.00	0.043	0.0019	0.00	0.032	0.0010	0.00	0.038	0.0014	0.00	0.043	0.0018
1	1	0	1	1	1.00	0.036	0.9963	0.00	0.034	0.0012	0.23	0.026	0.0554	0.06	0.031	0.0046	0.01	0.034	0.0012
0	2	0	1	1	0.40	0.019	0.1602	0.00	0.047	0.0022	0.00	0.018	0.0003	0.00	0.028	0.0008	0.00	0.043	0.0018
1	2	0	1	1	0.67	0.013	0.4447	0.00	0.039	0.0015	0.19	0.013	0.0377	0.09	0.026	0.0085	0.01	0.037	0.0015
0	1	1	1	1	0.67	0.023	0.4456	0.50	0.029	0.2520	0.38	0.024	0.1466	0.47	0.027	0.2215	0.50	0.029	0.2482
1	1	1	1	1	0.83	0.017	0.6924	0.33	0.028	0.1108	0.33	0.017	0.1105	0.33	0.024	0.1106	0.33	0.027	0.1108
0	2	1	1	1	0.50	0.017	0.2512	0.50	0.028	0.2481	0.22	0.018	0.0497	0.39	0.023	0.1557	0.48	0.027	0.2342
1	2	1	1	1	0.57	0.010	0.3258	0.33	0.030	0.1086	0.23	0.011	0.0525	0.28	0.020	0.0810	0.32	0.028	0.1040
0	1	2	1	1	0.50	0.016	0.2508	0.40	0.015	0.1618	0.36	0.015	0.1305	0.39	0.015	0.1549	0.40	0.015	0.1610
1	1	2	1	1	0.64	0.011	0.4052	0.33	0.018	0.1106	0.29	0.010	0.085	0.32	0.015	0.1036	0.33	0.017	0.1097
0	2	2	1	1	0.44	0.011	0.197	0.40	0.015	0.1603	0.27	0.012	0.0743	0.37	0.014	0.1356	0.40	0.015	0.1573
1	2	2	1	1	0.48	0.008	0.2275	0.33	0.021	0.1118	0.22	0.009	0.0477	0.29	0.016	0.0857	0.33	0.020	0.1081

Table A1. continued

Parameter settings					Frequentist model						Bayesian model								
					Unadjusted			*Adjusted for Z*			*Adjusted for Z, τ = 1000*			*Adjusted for Z, τ = 100*			*Adjusted for Z, τ = 10*		
β_{zu}	β_{xz}	β_{xu}	β_{yz}	β_{yu}	***Bias***	***SD***	***MSE***	***Bias***	***SD***	***MSE***	***Bias***	***SD***	***MSE***	***Bias***	***SD***	***MSE***	***Bias***	***SD***	***MSE***
0	1	0	2	1	1.00	0.04	1.0113	0.00	0.042	0.0017	0.00	0.031	0.001	0.00	0.036	0.0013	0.00	0.041	0.0017
1	1	0	2	1	1.67	0.042	2.7889	0.00	0.036	0.0013	0.23	0.022	0.0555	0.06	0.032	0.0049	0.01	0.036	0.0013
0	2	0	2	1	0.80	0.024	0.6362	0.00	0.048	0.0022	0.00	0.022	0.0005	0.00	0.031	0.0009	0.00	0.044	0.0019
1	2	0	2	1	1.11	0.017	1.239	0.00	0.037	0.0014	0.20	0.013	0.0386	0.09	0.023	0.0086	0.01	0.035	0.0013
0	1	1	2	1	1.00	0.032	1.008	0.50	0.027	0.2545	0.39	0.023	0.1496	0.47	0.026	0.2243	0.50	0.027	0.2507
1	1	1	2	1	1.33	0.025	1.7778	0.33	0.029	0.112	0.33	0.016	0.111	0.33	0.025	0.1116	0.33	0.028	0.112
0	2	1	2	1	0.84	0.020	0.6999	0.51	0.030	0.2569	0.22	0.018	0.0499	0.40	0.024	0.1602	0.49	0.029	0.2423
1	2	1	2	1	0.93	0.012	0.8652	0.33	0.029	0.1071	0.23	0.011	0.0532	0.28	0.019	0.0806	0.32	0.028	0.103
0	1	2	2	1	0.66	0.026	0.4382	0.40	0.015	0.1584	0.36	0.015	0.1284	0.39	0.015	0.1518	0.40	0.015	0.1577
1	1	2	2	1	1.00	0.016	1.0009	0.33	0.020	0.1112	0.29	0.012	0.0857	0.32	0.018	0.1042	0.33	0.020	0.1103
0	2	2	2	1	0.67	0.013	0.4446	0.40	0.016	0.1599	0.27	0.013	0.0744	0.37	0.015	0.1352	0.40	0.016	0.1568
1	2	2	2	1	0.76	0.011	0.5817	0.33	0.019	0.1114	0.22	0.009	0.0476	0.29	0.014	0.0852	0.33	0.018	0.1078
0	1	0	1	2	0.50	0.049	0.2501	-0.01	0.066	0.0044	0.00	0.047	0.0022	0.00	0.051	0.0026	-0.01	0.063	0.004
1	1	0	1	2	1.34	0.044	1.7869	0.01	0.057	0.0033	0.57	0.036	0.3208	0.22	0.046	0.049	0.03	0.055	0.0042
0	2	0	1	2	0.40	0.031	0.1633	0.00	0.070	0.0049	0.00	0.031	0.001	0.00	0.036	0.0013	0.00	0.059	0.0035
1	2	0	1	2	0.89	0.022	0.7868	0.00	0.056	0.0032	0.41	0.020	0.1711	0.26	0.029	0.0662	0.05	0.051	0.0051
0	1	1	1	2	1.00	0.032	1.0025	1.00	0.037	1.0028	0.72	0.035	0.5203	0.90	0.035	0.8033	0.99	0.037	0.9741
1	1	1	1	2	1.17	0.024	1.3603	0.67	0.038	0.4469	0.67	0.021	0.4455	0.67	0.030	0.4464	0.67	0.037	0.447
0	2	1	1	2	0.67	0.027	0.4476	1.00	0.041	1.0021	0.38	0.031	0.149	0.67	0.032	0.4461	0.94	0.039	0.8944
1	2	1	1	2	0.78	0.014	0.6121	0.66	0.038	0.4367	0.44	0.014	0.1977	0.54	0.022	0.2877	0.64	0.035	0.4059
0	1	2	1	2	0.83	0.019	0.695	0.80	0.020	0.6427	0.71	0.019	0.4988	0.78	0.02	0.605	0.80	0.02	0.6382
1	1	2	1	2	0.91	0.013	0.8261	0.67	0.023	0.4477	0.58	0.012	0.3318	0.64	0.019	0.4096	0.66	0.022	0.4426
0	2	2	1	2	0.67	0.012	0.4435	0.8	0.018	0.64	0.50	0.015	0.252	0.71	0.017	0.5023	0.79	0.018	0.6221
1	2	2	1	2	0.67	0.009	0.4437	0.67	0.021	0.4462	0.42	0.01	0.1747	0.56	0.015	0.3137	0.65	0.02	0.4249
0	1	0	2	2	0.99	0.059	0.9775	0.00	0.072	0.0051	-0.01	0.053	0.0028	0.00	0.057	0.0033	0.00	0.069	0.0047
1	1	0	2	2	2.00	0.051	4.0189	0.00	0.063	0.0039	0.56	0.033	0.3139	0.21	0.048	0.0465	0.03	0.061	0.0044
0	2	0	2	2	0.80	0.035	0.6375	0.00	0.074	0.0054	0.00	0.032	0.001	0.00	0.038	0.0015	0.00	0.063	0.0039
1	2	0	2	2	1.34	0.020	1.7868	0.01	0.053	0.0028	0.42	0.016	0.1756	0.26	0.026	0.07	0.06	0.047	0.0057
0	1	1	2	2	1.32	0.034	1.7485	0.99	0.041	0.9759	0.71	0.036	0.5027	0.88	0.038	0.7791	0.97	0.041	0.9476
1	1	1	2	2	1.67	0.024	2.7876	0.67	0.037	0.4469	0.67	0.018	0.4441	0.67	0.027	0.4457	0.67	0.035	0.4465
0	2	1	2	2	1.00	0.019	0.9931	1.00	0.043	0.999	0.38	0.022	0.1453	0.66	0.029	0.4413	0.94	0.04	0.8906
1	2	1	2	2	1.14	0.012	1.304	0.67	0.039	0.4491	0.45	0.011	0.1994	0.54	0.02	0.2937	0.65	0.036	0.4175
0	1	2	2	2	1.00	0.022	1.002	0.80	0.019	0.6394	0.70	0.019	0.4966	0.78	0.019	0.6019	0.80	0.019	0.6348
1	1	2	2	2	1.27	0.018	1.6236	0.67	0.026	0.4474	0.58	0.014	0.3309	0.64	0.022	0.4092	0.66	0.025	0.4425
0	2	2	2	2	0.89	0.016	0.7897	0.80	0.02	0.6395	0.50	0.02	0.2554	0.71	0.019	0.5045	0.79	0.02	0.6218
1	2	2	2	2	0.95	0.009	0.9051	0.67	0.025	0.4442	0.42	0.01	0.1744	0.56	0.017	0.3121	0.65	0.024	0.4229

τ indicate the precision of the prior distribution of the Z-Y relation in the Bayesian model.
Abbreviations: SD – standard deviation of the empirical distributions of the parameter estimates; MSE – mean squared error of the parameter estimates. See text in manuscript for details on simulation study.
